# *GREM1*, *FRZB* and *DKK1* mRNA levels correlate with osteoarthritis and are regulated by osteoarthritis-associated factors

**DOI:** 10.1186/ar4306

**Published:** 2013-09-19

**Authors:** Jeroen CH Leijten, Steffan D Bos, Ellie BM Landman, Nicole Georgi, Holger Jahr, Ingrid Meulenbelt, Janine N Post, Clemens A van Blitterswijk, Marcel Karperien

**Affiliations:** 1 Department of Developmental BioEngineering, MIRA Institute for Biomedical Technology and Technical Medicine, University of Twente, P.O. Box 217, 7500AE Enschede, the Netherlands; 2 Department of Molecular Epidemiology, Leiden University Medical Centre, Section of Molecular Epidemiology, Wassenaarseweg 72, 2333 AL Leiden, Netherlands; 3 Department of Orthopaedic Surgery, University Hospital RWTH Aachen, Pauwelsstraße 30, 52074 Aachen, Germany; 4 Department of Tissue Regeneration, University of Twente, Drienerlolaan 5, 7522NB Overijsse, Netherlands

## Abstract

**Introduction:**

Osteoarthritis is, at least in a subset of patients, associated with hypertrophic differentiation of articular chondrocytes. Recently, we identified the bone morphogenetic protein (BMP) and wingless-type MMTV integration site (WNT) signaling antagonists Gremlin 1 (*GREM1*), frizzled-related protein (*FRZB*) and dickkopf 1 homolog (*Xenopus laevis*) (*DKK1*) as articular cartilage’s natural brakes of hypertrophic differentiation. In this study, we investigated whether factors implicated in osteoarthritis or regulation of chondrocyte hypertrophy influence *GREM1*, *FRZB* and *DKK1* expression levels.

**Methods:**

*GREM1*, *FRZB* and *DKK1* mRNA levels were studied in articular cartilage from healthy preadolescents and healthy adults as well as in preserved and degrading osteoarthritic cartilage from the same osteoarthritic joint by quantitative PCR. Subsequently, we exposed human articular chondrocytes to WNT, BMP, IL-1β, Indian hedgehog, parathyroid hormone-related peptide, mechanical loading, different medium tonicities or distinct oxygen levels and investigated *GREM1*, *FRZB* and *DKK1* expression levels using a time-course analysis.

**Results:**

*GREM1*, *FRZB* and *DKK1* mRNA expression were strongly decreased in osteoarthritis. Moreover, this downregulation is stronger in degrading cartilage compared with macroscopically preserved cartilage from the same osteoarthritic joint. WNT, BMP, IL-1β signaling and mechanical loading regulated *GREM1*, *FRZB* and *DKK1* mRNA levels. Indian hedgehog, parathyroid hormone-related peptide and tonicity influenced the mRNA levels of at least one antagonist, while oxygen levels did not demonstrate any statistically significant effect. Interestingly, BMP and WNT signaling upregulated the expression of each other’s antagonists.

**Conclusions:**

Together, the current study demonstrates an inverse correlation between osteoarthritis and *GREM1*, *FRZB* and *DKK1* gene expression in cartilage and provides insight into the underlying transcriptional regulation. Furthermore, we show that BMP and WNT signaling are linked in a negative feedback loop, which might prove essential in articular cartilage homeostasis by balancing BMP and WNT activity.

## Introduction

A growing body of evidence suggests that hypertrophic differentiation of articular chondrocytes underlies the pathogenesis of osteoarthritis, at least in a subset of patients [1]. However, healthy articular cartilage is largely resistant to hypertrophic differentiation. In recent years many factors that are able to influence, or correlate with, the development of osteoarthritis have been revealed. These include, but are not limited to, bone morphogenetic proteins (BMPs) [2,3], canonical wingless**-**type MMTV integration site family members (WNTs) [4,5], Hedgehog [6], interleukins [7,8], parathyroid hormone-related peptide (PTHrP) [9] and the transcription factors HIF2A [10] and RunX2 [11]. Of these factors, BMPs [12], WNT [13], Indian hedgehog (IHH) [14], HIF2A [15] and RunX2 [16] have also been identified as prohypertrophic factors.

Regardless of the instigating factor, hypertrophic differentiation of chondrocytes induces a catabolic shift. Amongst others, IL-1β [17] and biomechanical stimulation, such as repetitive impulse loading [18], can also induce a catabolic shift. Additionally, tonicity might play a role in osteoarthritis, because it is significantly lower in osteoarthritic joints and is able to drive the expression of anabolic cartilage genes [19].

Healthy articular cartilage has an intrinsic mechanism that protects it from undergoing hypertrophic differentiation and subsequent catabolism [20]. Evidence suggests that articular cartilage is able to inhibit hypertrophic differentiation. For example, articular cartilage secretes soluble factors that inhibit hypertrophic differentiation of growth plate cartilage and chondrogenically differentiating mesenchymal stromal cells (MSCs) [21,22]. We recently identified the BMP and WNT antagonists Gremlin 1 (*GREM1*), frizzled-related protein (*FRZB*) and dickkopf 1 homolog (*Xenopus laevis*) (*DKK1*) as prime candidates for these articular cartilage secreted factors that inhibit chondrocyte hypertrophy [23]. Moreover, we have demonstrated that a SNP mutation in the *GREM1* gene associates with hip osteoarthritis [23].

Based on these observations, we hypothesized that the expression of *GREM1*, *FRZB* and *DKK1* is inversely correlated with osteoarthritis and their expression is influenced by established regulators of chondrocyte hypertrophy. In this study we have addressed this hypothesis by analyzing mRNA expression of *GREM1*, *FRZB* and *DKK1* in human cartilage biopsies and in primary human chondrocytes stimulated with factors that are able to influence, or correlate with, the development of osteoarthritis.

## Methods

### Patient material

The use of human material was approved by the medical ethical committee of the Leiden University Medical Center. Written informed consent was received from or on behalf of all patients, including next-of-kin for child patients. Healthy preadolescent articular cartilage was obtained from four patients between 9 and 14 years old that underwent amputation surgery with cartilage-unrelated etiologies. Healthy adult articular was obtained from three post-mortem donors (70.3 ± 11.2 years). Through the ongoing RAAK study [24] we sampled 23 donor joints (66.9 ± 9.9 years) with primary osteoarthritis during joint replacement surgery; cartilage specimens from areas visibly affected by the osteoarthritis process (osteoarthritis cartilage) and areas that appeared macroscopically intact (preserved cartilage) were taken for mRNA isolation and were analyzed pairwise.

### Cell isolation and cultivation

Macroscopically intact articular cartilage from osteoarthritic femoral condyles was obtained from patients undergoing total knee replacement to establish primary chondrocyte cultures. Bovine cartilage of the femoral condyle was obtained from a local abattoir. Chondrocytes were isolated by collagenase treatment and cultured as previously described [25]. Chondrocytes were used in passage 2 unless otherwise stated. One should note that expression of *GREM1*, *FRZB* and *DKK1* is not significantly altered between passage 0 and passage 2 chondrocytes (data not shown). Bone marrow-derived MSCs were isolated and cultured as described previously [26]. MG63 and Saos-2 were cultured in Dulbecco’s modified Eagle’s medium (Gibco, Grand Island, NY, USA) containing 10% heat-inactivated fetal bovine serum (Biowhittaker, Walkersville, MD, USA), 100 U/ml penicillin (Gibco) with 100 mg/ml streptomycin (Gibco).

### Oxygen levels

Freshly isolated human chondrocytes were seeded at 2,500 cells/cm^2^ and cultured under conventional normoxic culture conditions (21% oxygen) or under hypoxic culture conditions (2.5% oxygen) using a hypoxia incubator (proox model C21; Biospherix, Redfield, NY, USA). Cells were cultured until 95% confluency was reached.

### Tonicity

Chondrocytes were seeded at 7,500 cells/cm^2^, expanded in culture medium that was adjusted to either 280 or 380 mOsm, which approximates the osmolarity found in the synovial fluid of an osteoarthritic and healthy joint respectively [27]. We therefore refer to 280 mOsm as being hypotonic. The osmolarity of the culture medium was adjusted with sodium chloride and reseeded at 20,000 cells/cm^2^ as previously described [19]. After 24 hours, 0 or 500 ng/ml calcineurin inhibitor FK506 was supplemented to the culture medium. FK506 inhibits calcineurin-dependent NFAT signaling, which has been described to mediate tonicity-induced cell signaling in chondrocytes [19]. After an additional 6 days the chondrocytes were lysed for gene expression analysis.

### Mechanical stimulation

A medium suspension of passage 2 human chondrocytes was mixed in a 1:1 ratio with liquefied ultrapure agarose (Invitrogen, Carlsbad, CA, USA) and loaded into a stainless steel bioreactor to create four 70 μl constructs of two percent agarose containing 10×10^6^ cells/ml. The insert, loaded with four constructs, was installed in a custom-build bioreactor [28]. Using a custom-designed compression plate, two out of four constructs were mechanically loaded with 0.5 MPa, while the other two constructs remained unloaded. Compression was applied in a cyclical fashion with a frequency of 0.33 Hz with a loading phase of 50%. During 48 hours, the loaded samples were either cyclically compressed without interruption (constant loading) or were unloaded for 1 hour after being cyclically compressed for 1 hour (intermittent loading).

### Recombinant protein and compound stimulation

All cell types were seeded at 5,000 cells/cm^2^, grown to 95% confluency and subsequently exposed to a single dose of recombinant proteins. In all stimulation experiments, primary human chondrocytes of intact osteoarthritic cartilage, bovine chondrocytes of healthy articular cartilage, human bone marrow-derived MSCs or the cell lines MG63 and Saos-2 were used at passage 2. Cells received no medium refreshment after stimulation had occurred and were cultured up to 96 hours unless otherwise stated.

Human chondrocytes were exposed to 4, 20, 100 or 200 ng/ml recombinant human BMP2 (catalogue number 355-BM-010; R&D Systems, Minneapolis, MN, USA), 100 ng/ml recombinant human WNT3A (catalogue number 5036-WN-010; R&D Systems), 100 ng/ml recombinant human *DKK1* (catalogue number 5439-DK-010; R&D Systems), 3, 10 or 30 nM GSK3β inhibitor GIN [29], 0.3, 1 or 3 μM canonical WNT inhibitor PKF115-584 [30], 10 or 100 ng/ml recombinant human (catalogue number IL1B 201-LB-005; R&D Systems), 10 μM hedgehog signaling blocker cyclopamine (catalogue number BML-GR334-0001; Enzo Life Sciences, Farmingdale, NY, USA), 2.5 μg/ml recombinant IHH (catalogue number 1705-HH-025; R&D Systems) or 5×10^–7^ M recombinant human PTHrP (catalogue number SRP4651-50; Sigma Aldrich, St Louis, MO, USA). Second-passage human and bovine chondrocytes, second-passage human MSCs, MG63 and Saos-2 were stimulated with 10 nM GIN and 100 ng/ml recombinant human WNT3A.

### Quantitative real-time RT-PCR

At designated time points, cells were washed with PBS and lysed using trizol reagent (Invitrogen). Total RNA isolation, cDNA synthesis and gene expression analysis were performed as described previously [26]. Gene expression is reported as the relative fold-change between treated samples and untreated controls and is normalized to 0 hours post treatment unless stated otherwise. Primer sequences are available on our website [31].

### Viability

Passage 2 human chondrocytes were seeded at 3,000 cells/cm^2^. Upon reaching 95% confluency the chondrocytes were treated with 3, 10, 30 nM GIN, 0.3, 1, 3 μM PKF115-584, 100 ng/ml *DKK1* or 100 ng/ml WNT3A. After 48 hours the total metabolic activity of the cells was measured using Alamar blue (Invitrogen) according to the manufacturer’s protocol.

### Statistical analysis

Statistical differences between experimental treatments were analyzed using Student’s t test or one-way analysis of variance. Each group consisted of three different donors, each measured at least in triplicate. Correlation between the expression of different genes was calculated using Pearson correlation. Statistical significance was set to *P <*0.05.

## Results

### ***GREM1***, ***FRZB*** and ***DKK1*** mRNA levels are decreased in osteoarthritic cartilage

**Figure 1:**
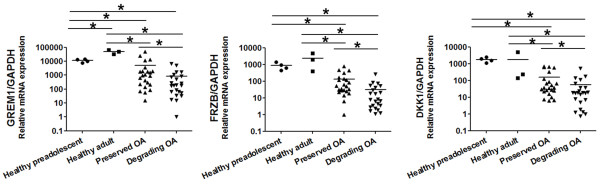
***GREM1*, *FRZB *and *DKK1 *expression in healthy cartilage and osteoarthritic cartilage.** Relative *GREM1*, *FRZB* and *DKK1* mRNA expression levels of paired specimens of macroscopically preserved and degenerating osteoarthritic cartilage from a single osteoarthritic joint (*n* = 23) were assessed by quantitative PCR and were compared with healthy preadolescent (*n* = 4) and healthy adult (*n* = 3) articular cartilage specimen. Data expressed as fold-change relative to the specimen with the mRNA expression level on a log scale. *P <0.05. *DKK1*, dickkopf 1 homolog (*Xenopus laevis*); *FRZB*, frizzled-related protein; GADPH, glyceraldehyde 3-phosphate dehydrogenase; *GREM1*, Gremlin 1; OA, osteoarthritis.

Osteoarthritic cartilage is characterized by evidence of increased hypertrophic differentiation in at least a subset of patients [32]. Recently we reported that *FRZB*, *GREM1* and *DKK1* function as natural brakes of the hypertrophic differentiation of articular cartilage [23]. Based on their proposed role, we hypothesized that the expression of these genes was decreased in osteoarthritic cartilage. *GREM1*, *FRZB* and *DKK1* mRNA expression levels were therefore determined in paired specimens of macroscopically relatively preserved and degenerating osteoarthritic cartilage collected from a single osteoarthritic joint for 23 patients, healthy preadolescent cartilage and healthy adult articular cartilage. Healthy preadolescent and healthy adult articular cartilage both express these three genes at significantly higher levels than preserved and degrading osteoarthritic cartilage. Moreover, *GREM1*, *FRZB* and *DKK1* mRNA is significantly lower in degrading cartilage compared with macroscopically preserved cartilage (Figure 1) by 5.86-fold, 4.34-fold and 2.83 fold respectively (in Additional file 1: Figure S1A). In addition, we demonstrated that this decrease is reproducible in almost all tested patients, with the exception of the patients with the lowest *GREM1*, *FRZB* and *DKK1* mRNA expression levels. Finally, we demonstrated that the expression of all three genes was positively correlated with each other (in Additional file 1: Figure S1B).

### BMP2 enhances transcription of WNT antagonist ***FRZB*** and ***DKK1***

**Figure 2:**
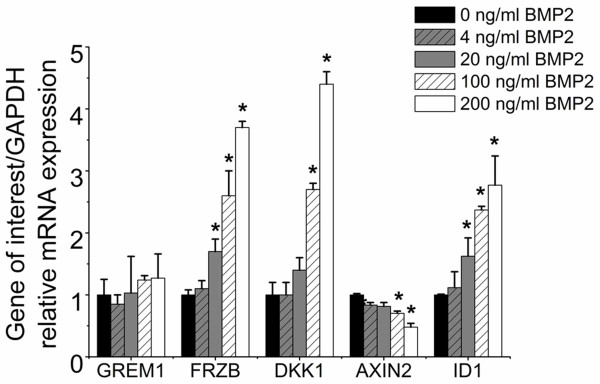
**Effects of bone morphogenetic protein signaling on the mRNA expression of *GREM1*, *FRZB *and *DKK1*.** Chondrocytes were stimulated for 48 hours with different concentrations of BMP2 ranging from 0 to 200 ng/μl. Effects on gene expression of *GREM1*, *FRZB*, *DKK1*, *AXIN2* and *DKK1* were analyzed using quantitative PCR. Data expressed as fold-change relative to control and represents the mean of three donors ± standard deviation. *P <0.05 compared with 0 ng/ml BMP2. BMP2, bone morphogenetic protein 2; *DKK1*, dickkopf 1 homolog (*Xenopus laevis*); *FRZB*, frizzled-related protein; GADPH, glyceraldehyde 3-phosphate dehydrogenase; *GREM1*, Gremlin 1.

Human chondrocytes were stimulated with a single pulse of BMP2 for up to 48 hours to investigate its effect on *GREM1*, *FRZB* and *DKK1* mRNA levels. Functionality of the recombinant protein was demonstrated by a dose-dependent induction of the BMP target gene *ID1*. *GREM1* mRNA levels remained unaltered compared with untreated chondrocytes after 48 hours. However, the expression of the WNT antagonists *FRZB* and *DKK1* were significantly upregulated in a dose-dependent manner (Figure 2). This suggests that BMPs were able to induce inhibition of canonical WNT signaling in a dose-dependent manner. Indeed, the WNT target gene *AXIN2* was downregulated in a dose-dependent manner after BMP2 treatment.

### Canonical WNT signaling downregulates ***GREM1***, ***FRZB*** and ***DKK1*** expression

**Figure 3:**
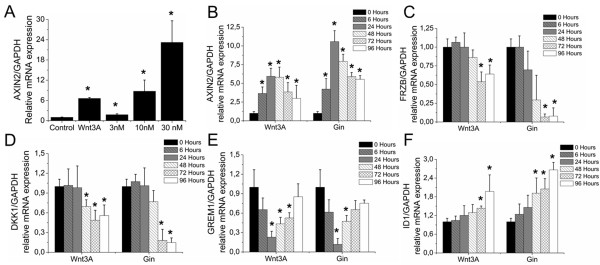
**Effect of canonical WNT signaling on the mRNA expression of *GREM1*, *FRZB *and *DKK1*. (A)** Primary human chondrocytes were exposed to 100 ng/ml WNT3A or three different concentrations of GIN. After 48 hours, *AXIN2* mRNA expression was analyzed by quantitative PCR. **(B)** to **(F)** Chondrocytes were exposed to a single dose of 10 nM GIN or 100 ng/ml WNT3A. At indicated time points, mRNA expression was analyzed by quantitative PCR of *AXIN2***(B)**, *FRZB***(C)**, *DKK1***(D)**, *GREM1***(E)** and *ID1***(F)**. Data expressed as fold-change relative to untreated time-point-matched control and represents the mean of three donors ± standard deviation. *P <0.05 compared with unstimulated cells **(A)** or 0 hours of stimulation **(B)** to **(F)**. *DKK1*, dickkopf 1 homolog (*Xenopus laevis*); *FRZB*, frizzled-related protein; GADPH, glyceraldehyde 3-phosphate dehydrogenase; *GREM1*, Gremlin 1; WNT, wingless-type MMTV integration site.

Chondrocytes were cultured in the presence or absence of either 100 ng/ml recombinant WNT3A, or a dose range of the GSK3*β* inhibitor GIN, which activates canonical WNT signaling. Both WNT3A and GIN dose-dependently upregulated *AXIN2* mRNA expression 48 hours after stimulation (Figure 3A). None of the conditions showed any signs of cytotoxicity as determined by phenotypical appearance and metabolic activity of the cells (Additional file 2).

Chondrocytes were then cultured in the presence or absence of 100 ng/ml WNT3A or 10 nM GIN up to 96 hours. Both GIN and WNT3A induced canonical WNT signaling evidenced by an increase in AXIN2 mRNA expression. The effect was first detected after 6 hours and peaked between 24 and 48 hours post stimulation (Figure 3B). *FRZB* and *DKK1* mRNA levels started to decrease 48 hours after stimulation and were significantly lower after 72 and 96 hours compared with untreated samples. This suggested that activation of WNT signaling resulted in the downregulation of WNT antagonists (Figure 3C, D). Activation of canonical WNT signaling transiently decreased *GREM1* mRNA expression with lowest levels of mRNA expression 24 hours after treatment, after which the expression levels gradually returned to control levels (Figure 3E).

Additionally, we investigated the effects of enhanced WNT signaling on the mRNA levels of *CHRD* and *CHRDL2*, two BMP antagonists that have been suggested to play an inhibitory role in hypertrophic differentiation of chondrocytes [33,34]. Activation of canonical WNT signaling reduced *CHRD* and *CHRDL2* mRNA levels with a maximal effect after 72 hours (in Additional file 3: Figure S3A, B). This suggested that activation of canonical WNT signaling might be able to influence BMP signaling by decreasing the expression of BMP antagonists. Indeed, mRNA levels of the established BMP target gene *ID1* increased upon stimulation of canonical WNT signaling. This increase was preceded by a decrease in BMP antagonists’ gene transcription (Figure 3F).

### Canonical WNT signaling regulates ***GREM1***, ***FRZB*** and ***DKK1*** mRNA levels in bovine chondrocytes, MG63, SAOS2, and human mesenchymal stromal cells

As activation of canonical WNT signaling is correlated with a catabolic response in cartilage, at least in animal models, it is paramount for joint homeostasis that WNT signaling is tightly controlled. Typically, activation of critical pathways is accompanied by subsequent activation of negative feedback loops reducing pathway activity. Surprisingly, activation of canonical WNT signaling in primary human chondrocytes resulted in decreased *FRZB* and *DKK1* mRNA levels (Figure 3C, D). We therefore tested whether this downregulation was restricted to articular chondrocytes or was a general response across different cell types. Bovine chondrocytes, MG63s, SAOS-2 and MSCs were exposed to 100 ng/ml WNT3A or 10 nM GIN for 48 hours. Comparable with human chondrocytes, bovine chondrocytes downregulated *FRZB* and *DKK1* mRNA levels after activation of canonical WNT signaling. In contrast, MG63 and SAOS-2 did not respond to GIN with changes in expression of *FRZB* and *GREM1,* respectively (Additional file 4). Like chondrocytes, human bone marrow-derived MSCs demonstrated a decrease in *FRZB* and *DKK1* mRNA levels upon stimulation of canonical WNT signaling. In contrast to human chondrocytes, *GREM1* mRNA expression was upregulated by activating WNT signaling. Together this suggested that the response to canonical WNT signaling stimulation with regards to the mRNA expression levels of WNT and BMP antagonists is cell type dependent, but is conserved between species in articular chondrocytes.

### Inhibition of canonical WNT signaling induces mRNA expression of ***GREM1***, ***FRZB*** and ***DKK1***

We next investigated the effect of inhibiting canonical WNT signaling on the mRNA expression levels of *GREM1*, *FRZB* and *DKK1* using 100 ng/ml WNT antagonist *DKK1* or 0.3, 1 or 3 μM canonical WNT inhibitor PKF115-584. Treatment of human chondrocytes for 48 hours with either WNT inhibitor significantly reduced *AXIN2* mRNA levels, except for 0.3 μM PKF115-584 (in Additional file 5: Figure S5A). Treatment with 1 or 3 μM PKF115-584 reduced the chondrocyte’s metabolic activity and chondrocytes treated with 3 μM PKF115-584 showed phenotypical signs of stress (Additional file 2). A concentration of 1 μM PKF115-584 was therefore selected for further experimentation.

Treatment of chondrocytes up to 96 hours with a single dose of 100 ng/ml *DKK1* or 1 μM PKF115-584 resulted in a progressive decrease in *AXIN2* mRNA levels, which became statistically significant between 72 and 96 hours post treatment (in Additional file 5: Figure S5B). In contrast, *FRZB* and *DKK1* mRNA levels steadily increased over time, which became significant between 24 and 48 hours post exposure (in Additional file 5: Figure S5C, D). Treatment with *DKK1* or PKF115-584 increased *GREM1* mRNA levels and this coincided with a subsequent decrease in *ID1* mRNA levels (in Additional file 5: Figure S5E, F). Taken together, these data suggested that in human chondrocytes the *GREM1*, *FRZB* and *DKK1* mRNA levels were inversely related to the activity of canonical WNT signaling.

### Effects of IL-1β stimulation on ***GREM1***, ***FRZB*** and ***DKK1*** mRNA expression

**Figure 4:**
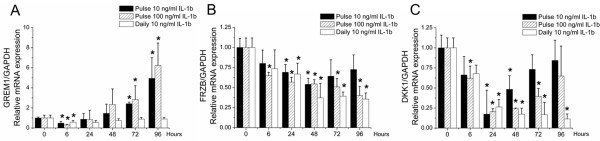
**Effects of IL-1β-mediated signaling on mRNA expression of *GREM1*, *FRZB *and *DKK1*.** Chondrocytes received a single dose of 10 or 100 ng/ml IL-1β, or a daily medium refreshment containing 10 ng/ml IL-1β. Chondrocytes were stimulated up to 96 hours and mRNA expression was analyzed by quantitative PCR at the indicated time points for *GREM1***(A)**, *FRZB***(B)**, and *DKK1***(C)**. Data expressed as fold-change relative to untreated time point-matched control and represent the mean of three donors ± standard deviation. *P <0.05 compared with unstimulated cells. *DKK1*, dickkopf 1 homolog (*Xenopus laevis*); *FRZB*, frizzled-related protein; GADPH, glyceraldehyde 3-phosphate dehydrogenase; *GREM1*, Gremlin 1.

Local injection of IL-1β into mouse knee joints resulted in the destabilization of joint homeostasis by inducing a catabolic shift in the articular cartilage [35]. We therefore investigated the effect of IL-1β on *GREM1*, *FRZB* and *DKK1* mRNA levels. Chondrocytes were stimulated with either a single dose of 10 or 100 ng/ml IL-1β or with a daily repeated dose of 10 ng/ml IL-1β. Upon exposure to a single dose of IL-1β, *GREM1* mRNA levels decreased at 6 hours followed by a steady increase in mRNA expression, which became significantly higher than untreated samples after 72 hours. Interestingly, repeated treatment with of IL-1β decreased *GREM1* mRNA expression after 6 hours, which returned to baseline after 24 hours (Figure 4A). *FRZB* mRNA levels were dose-dependently downregulated after exposure to IL-1β. Daily treatment with 10 ng/ml was as effective as a pulse treatment with 100 ng/ml. *DKK1* mRNA levels decreased after stimulation with IL-1β. This downregulation was transient with a single dose but persistent with a daily dose of IL-1β (Figure 4B, C).

### Effects of physiological factors on ***GREM1***, ***FRZB*** and ***DKK1*** mRNA transcription

Many physiological factors influence the homeostasis of articular cartilage. To determine whether such stimuli have an effect on the mRNA expression levels of *GREM1*, *FRZB* and *DKK1*, chondrocytes were exposed to, for example, differing mechanical compression, oxygen tension and tonicity.

**Figure 5:**
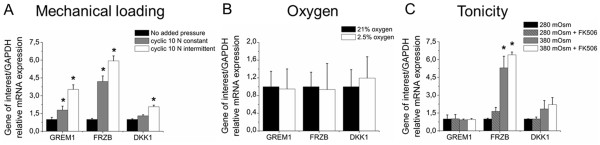
**Effects of physiological factor-mediated signaling on mRNA expression of *GREM1*, *FRZB *and *DKK1*.** Effects of mechanical loading, oxygen level and medium tonicity mediated signaling on mRNA expression of *GREM1*, *FRZB* and *DKK1*. Chondrocytes encapsulated in a hydrogel received no, intermittent or constant cyclical mechanical loading of 0.5 MPa with a frequency of 0.33 Hz and a loading phase of 50% **(A)**. Chondrocytes were exposed to normoxic or hypoxic culture conditions **(B)**, or were cultured in media with different tonicity with or without FK506 **(C)**. All conditions were analyzed for the mRNA expression of *GREM1*, *FRZB* and *DKK1* by quantitative PCR after 48 hours. Data expressed as fold-change relative to control and represent the mean of three donors ± standard deviation. *P <0.05 compared with unloaded samples or samples containing medium of 280 mOsm. *DKK1*, dickkopf 1 homolog (*Xenopus laevis*); *FRZB*, frizzled-related protein; GADPH, glyceraldehyde 3-phosphate dehydrogenase; *GREM1*, Gremlin 1.

Chondrocytes encapsulated in three-dimensional hydrogels were cultured in the presence or absence of cyclic loading of the construct with 0.5 MPa with a frequency of 0.33 Hz, which was either continuously or intermittently applied over a culture period of 48 hours. Both continuous and intermittent loading significantly elevated *GREM1* and *FRZB* mRNA levels compared with unloaded chondrocytes. *DKK1* mRNA levels were only significantly increased after intermittent loading of the construct (Figure 5A). Interestingly, intermittent loading was more effective in upregulating *GREM1*, *FRZB* and *DKK1* mRNA expression than continuous loading.

Articular cartilage predominantly persists in a continuous state of hypoxia. Relief of this hypoxic stress is able to stimulate hypertrophic differentiation of hyaline cartilage [36]. Chondrocytes were therefore cultured under hypoxic (2.5%) or normoxic (21%) conditions. No detectable changes in *GREM1*, *FRZB* and *DKK1* mRNA levels were observed between normoxic and hypoxic culture conditions (Figure 5B).

Osteoarthritis is associated with a decrease in tonicity of the synovial fluid and cartilage [27]. We therefore investigated the effect of tonicity on the mRNA levels of *GREM1*, *FRZB* and *DKK1*. Tonicity did not detectably affect *GREM1* mRNA levels (Figure 5C). In contrast, tonicity tended to increase *DKK1* mRNA levels and significantly increased *FRZB* mRNA levels. This effect was NFAT independent because FK506, which indirectly inhibits NFAT nuclear translocation, had no significant effect on *GREM1*, *FRZB* and *DKK1* mRNA levels.

### Effects of PTHrP, IHH and cyclopamine on ***GREM1***, ***FRZB*** and ***DKK1*** mRNA expression

PTHrP and IHH expression in articular cartilage is correlated with osteoarthritis [6,9]. In addition, PTHrP and IHH critically regulate the pace of hypertrophic differentiation in growth plate cartilage in a negative feedback loop. As *GREM1*, *FRZB* and *DKK1* were able to inhibit hypertrophic differentiation in articular cartilage and mitigated longitudinal bone growth in explanted mouse fetal long bones [23], we investigated whether PTHrP and IHH were able to influence their mRNA expression. Chondrocytes were cultured up to 96 hours in the presence or absence of PTHrP, IHH and the hedgehog signaling blocker cyclopamine. *GREM1* mRNA expression remained unchanged when stimulated with PTHrP, tended to transiently decrease after stimulation with IHH and was significantly increased by cyclopamine after 72 and 96 hours (in Additional file 6: Figure S6A). PTHrP nor IHH affected *FRZB* mRNA levels. Cyclopamine tended to decrease *FRZB* mRNA expression but this did not reach significance (in Additional file 6: Figure S6B). In contrast, *DKK1* mRNA expression was significantly increased by cyclopamine and PTHrP treatment from 24 and 72 hours, respectively, but not by IHH (in Additional file 6: Figure S6C). The expression of established hedgehog and PTHrP target genes was used to verify the biological activity of each stimulus. *GLI1* mRNA levels increased in the presence of IHH (in Additional file 6: Figure S6D) and decreased in the presence of cyclopamine (in Additional file 6: Figure S6E). *CCND1* mRNA levels increased in the presence of PTHrP (in Additional file 6: Figure S6F). Taken together, this suggested that inhibition, but not activation, of IHH upregulated *GREM1* and *DKK1* mRNA levels.

## Discussion

Recently we have reported that *GREM1*, *FRZB* and *DKK1* are enriched in articular cartilage compared with other hyaline cartilage types and act as potent inhibitors of hypertrophic differentiation [23]. Moreover, we demonstrated an association between a genetic variation (SNP rs12593365) in a genomic control region of *GREM1* and radiographic osteoarthritis of the hip. Based on these and other data we provided evidence that these BMP and WNT antagonists are important regulators of articular cartilage homeostasis by preventing hypertrophic differentiation of chondrocytes [23]. Since osteoarthritis is associated with deregulated hypertrophic differentiation in at least a subset of patients, we hypothesized that *GREM1*, *FRZB* and *DKK1* mRNA expression levels are downregulated in osteoarthritis. In this study we report that the expression of *GREM1*, *FRZB* and *DKK1* mRNA was strongly decreased in osteoarthritic cartilage compared with healthy cartilage and was also decreased in degrading osteoarthritic cartilage compared with macroscopically preserved cartilage from the same osteoarthritic joint. In addition, we report on the effects of biochemical and biophysical stimuli associated with chondrocyte hypertrophy on *GREM1*, *FRZB* and *DKK1* mRNA expression. Although the enzymatic isolation of the chondrocytes may have affected their gene expression levels, they were directly in line with our hypothesis. Furthermore, our claims are furthermore supported by recent observations demonstrating that osteophytic cartilage, which is prone to undergo endochondral ossification, has significantly less expression of *GREM1* and *FRZB* compared with permanent articular cartilage [37].

*GREM1*, *FRZB* and *DKK1* are secreted soluble antagonists. *FRZB* and *DKK1* are WNT antagonists and *GREM1* is a BMP antagonist. *GREM1* is also able to inhibit WNT signaling via unknown indirect mechanisms [38] and BMP signaling is able to repress WNT signaling [39]. Conversely, WNT signaling is also able to repress BMP signaling [40]. Activation of WNT signaling is well known to inhibit fibroblastic growth factor-dependent BMP repression [41]. Indeed, increased canonical WNT signaling resulted in increased BMP/SMAD signaling [42]. Although the crosstalk between BMP and WNT signaling is suggested to involve SMAD4 and MAPK p38, the exact mechanism has remained largely unknown [43].

**Figure 6:**
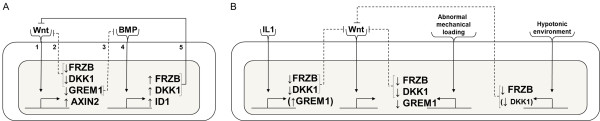
**Preliminary working model of WNT and BMP signaling feedback loop and perturbation by osteoarthritis factors. (A)** WNT and BMP signaling reciprocally regulates the transcription of other antagonists. Exposure of WNT agonists leads to activation of WNT signaling [1]. This activation results in the downregulation of WNT antagonists (for example, *FRZB* and *DKK1*), leading to less inhibition of WNT signaling [2]. Additionally, BMP antagonists (for example, *GREM1*) are downregulated, leading to less inhibition of BMP signaling [3]. Stronger BMP signaling results in the upregulation of WNT antagonists [4], establishing a negative feedback mitigating WNT signaling [5]. This feedback loop allows for tight control of both BMP and WNT signaling in articular cartilage contributing to homeostasis. **(B)** Established factors that influence cartilage homeostasis also perturb this feedback loop. IL-1β, lack of mechanical stimulation and tonicity all decrease the mRNA levels of WNT and BMP antagonists, possibly resulting in a reset of the feedback loop, and contributing to the loss of cartilage homeostasis. BMP, bone morphogenetic protein; *DKK1*, dickkopf 1 homolog (*Xenopus laevis*); *FRZB*, frizzled-related protein; GADPH, glyceraldehyde 3-phosphate dehydrogenase; *GREM1*, Gremlin 1; WNT, wingless-type MMTV integration site.

Understanding the crosstalk between transforming growth factor beta/BMP and WNT signaling is desired because it plays important roles in the formation of several tissues, including bone, cartilage and intestinal epithelium [42,43]. While in previous studies the interplay is mainly characterized by activation of each other’s ligands, in our study we provide evidence that antagonists may also play a central role in the crosstalk. We demonstrated in chondrocytes that this crosstalk is mediated, at least partially, via reciprocal transcriptional control of each other’s antagonists. Consequently, our data suggest that the crosstalk between transforming growth factor beta/BMP and WNT signaling might act as a feedback loop that balances the activity of both pathways (Figure 6A). Specifically, activation of WNT signaling downregulates the expression of genes encoding BMP antagonists and is associated with the upregulation of BMP signaling. In turn, activation of BMP signaling results in increased expression of genes encoding WNT antagonists, which is associated with decreased WNT signaling.

Stringent control over the WNT pathway’s activity is paramount for articular cartilage homeostasis because both exacerbated and repressed signaling results in an osteoarthritis-like phenotype, at least in animal models [44,45]. Perturbation of feedback loops that control the activity of WNT signaling would therefore allow for the disturbance of the natural homeostasis. Factors associated with disturbed joint homeostasis include, amongst others, IL-1β, abnormal mechanical loading and hypotonicity. Although evidence suggests that short exposure to IL-1β results in minor joint inflammation without permanent joint destruction, continuous exposure to IL-1β results in joint degradation that bears striking resemblance with osteoarthritis [7,34]. Interestingly, IL-1β activates WNT signaling via a currently unknown mechanism [46]. Excessive mechanical loading of the joint induces the expression of IL-1β and catabolic proteins [47]. Interestingly, reduced joint loading by, for example, immobilization also results in increased catabolism via the upregulation of matrix metalloproteinases and aggrecanases [48]. In contrast, loading within the physiological range inhibits the expression of catabolic genes and shows a chondroprotective effect in the presence of IL-1β [49]. Additionally, mechanical loading is able to regulate the activity of WNT signaling via a currently unknown mechanism [50]. Tonicity is able to regulate the expression of interleukins including IL-1β [51]. However, its effect on WNT signaling has remained largely uninvestigated. In this study, we present data implying that IL-1β, lack of mechanical loading and hypotonicity downregulate the expression of the genes encoding hypertrophic differentiation inhibiting proteins including *GREM1*, *FRZB* and *DKK1* (Figure 6B).

Our data suggest that these factors might be able to perturb the balance between BMP and WNT signaling by influencing the expression of both WNT and BMP antagonists in a manner that cannot be sequestered via their regular feedback loops. Consequently, it is tempting to speculate that these factors may contribute to an osteoarthritic-like phenotype, at least partially, via their ability to disturb the balance between WNT and BMP signaling. Although additional research is needed to sustain such a claim, recent evidence demonstrated that the addition of WNT and BMP antagonist sclerostin was able to prevent an IL-1β-induced osteoarthritis-like phenotype [52].

Stimulation with BMPs, WNTs, IL-1, IHH, PTHrP, oxygen concentrations, change in tonicity and mechanical loading may also influence the expression of WNT and BMP antagonists other than *GREM1*, *FRZB* and *DKK1*. Furthermore, such stimulation might also influence the expression of WNT and BMP agonists, at least at the mRNA level. Moreover, it remains to be noted that the current study is indeed limited to mRNA expression and not protein abundance. Nonetheless, clinical studies have shown that *DKK1* and *FRZB* protein expression in serum and/or synovial fluid are expressed at significantly different levels in patients with osteoarthritis or rheumatoid arthritis compared with control [53,54]. Similarly decreased *DKK1* levels are found in synovial fluid of animal models of osteoarthritis. Interestingly, *DKK1* supplementation has recently been shown to protect from experimental osteoarthritis [55]. Lastly, several other BMP and WNT related proteins have been indicated as being either protective or destructive for articular cartilage [56,57]. These observations are in line with our hypothesis and emphasize that stringent control over *DKK1*, *FRZB* and *GREM1* expression is required to maintain cartilage homeostasis by preventing hypertrophic chondrocyte differentiation and subsequent catabolism.

## Conclusion

The current study demonstrates that the mRNA expression of *GREM1*, *FRZB* and *DKK1* is inversely correlated with the level of cartilage degeneration in osteoarthritis. Moreover, the expression of these regulators of chondrocyte hypertrophy can be influenced by regulators of chondrocyte hypertrophy. Together, this provides a deeper understanding of chondrocyte behavior, cartilage homeostasis and osteoarthritis.

## Abbreviations

AXIN2: Axin-related protein; BMP: Bone morphogenetic protein; CHRD: Chordin; CHRDL2: Chordin-like 2; DKK1: Dickkopf 1 homolog (*Xenopus laevis*); FRZB: Frizzled-related protein; GREM1: Gremlin 1; IHH: Indian hedgehog; IL: Interleukin; MSC: Mesenchymal stromal cell; PBS: Phosphate-buffered saline; PTHrP: Parathyroid hormone-related peptide; RT: Reverse transcriptase; PCR: Polymerase chain reaction; SNP: Single nucleotide polymorphism; WNT: Wingless-type MMTV integration site.

## Competing interests

The authors declare that they have no competing interests.

## Authors’ contributions

JCHL conceived the study. SDB and IM collected and analyzed the gene expression of adult patients with either osteoarthritis or rheumatoid arthritis. EBML, NG and HJ performed and analyzed the IL-1β, oxygen tension and tonicity experiments. JCHL performed and analyzed the remaining gene expression experiments. JNP, CAvB and MK participated in the study’s design, coordination and data analysis. JCHL and MK drafted the manuscript. SDB, EBML, NG, HJ, IM, JNP and CAvB aided in revising the manuscript’s intellectual content. All authors read and approved the final manuscript.

## Supplementary Material

Additional file 1: Figure S1 Showing correlation of *GREM1*, *FRZB* and *DKK1* mRNA expression in preserved and degrading cartilage from the same osteoarthritic joint (OA). (A) *GREM1*, *FRZB* and *DKK1* mRNA levels were determined in preserved and degenerated cartilage from the same joint. All three antagonists are significantly less expressed in degenerated cartilage compared with preserved cartilage of the same joint (*n* = 23). Broken line indicates the cartilage specimen that belongs to the same donor. (B) *GREM1*, *FRZB* and *DKK1* were investigated on correlation of mRNA expression. Correlation was determined using Pearson correlation. GADPH, glyceraldehyde 3-phosphate dehydrogenase. Click here for file

Additional file 2: Figure S2 Showing that cytotoxicity was phenotypically investigated by assessing chondrocyte morphology. Cells were exposed to DMSO, 100 ng/nl WNT3A, and 100 ng/ml *DKK1* (A), a concentration range of GIN (B) and a concentration range of PKF115-584 (C). A representative picture of each condition over time is shown. To further investigate cytotoxicity, the metabolic activity of chondrocytes exposed to the different conditions was determined using Alamar Blue quantification (D). Data represent the mean of three donors ± standard deviation. DMSO, dimethylsulfoxide. Click here for file

Additional file 3: Figure S3 Showing that WNT signaling decreases chordin and chordin-like 2 mRNA expression in primary human chondrocytes. Gene expression was measured by quantitative PCR. Chondrocytes were exposed to a single dose of 10 nM GIN, 100 ng/ml WNT3A (A), (B), 1 μM PKF115-584 or 100 ng/ml *DKK1* (C), (D) for up to 72 hours and analyzed for the gene transcription of *CHRD* (A, C) and *CHRDL2* (B, D). Data represent the mean of three donors ± standard deviation. *P <0.05 compared with untreated time point-matched controls. GADPH, glyceraldehyde 3-phosphate dehydrogenase. Click here for file

Additional file 4: Figure S4 Showing effects of activation of canonical WNT signaling on the mRNA expression of *GREM1*, *FRZB* and *DKK1* in cells other than human chondrocytes. A single dose of 100 ng/ml WNT3A or 10 nM GIN was added to the culture media of bovine chondrocytes, the MG63 cell line, the SAOS-2 cell line and human bone marrow-derived MSCs. After 48 hours the gene transcription levels of *GREM1* (A), *FRZB* (B) and *DKK1* (C) were analyzed. Data represent the mean of three independent experiments (bovine chondrocytes, MG63 and SAOS-2) or two donors (MSCs) ± standard deviation. *P <0.05 compared with untreated time point-matched controls. GADPH, glyceraldehyde 3-phosphate dehydrogenase. Click here for file

Additional file 5: Figure S5 Showing effect of canonical WNT signaling on the mRNA expression of *GREM1*, *FRZB* and *DKK1*. Primary human chondrocytes were exposed to 100 ng/ml WNT3A or three different concentrations of PKF115-584 (115). After 48 hours *AXIN2* mRNA expression was analyzed by quantitative PCR. (A) Primary human chondrocytes were then exposed to 1 μM PKF115-584 or 100 ng/ml *DKK1* for up to 96 hours. At indicated time points, gene expression was analyzed by quantitative PCR of *AXIN2* (B), *FRZB* (C), *DKK1* (D), *GREM1*, (E) and *ID1* (F). Data expressed as fold-change relative to control and represents the mean of three donors ± standard deviation. *P <0.05 compared with untreated time point-matched controls (G) or 0 hours of stimulation (A to F). GADPH, glyceraldehyde 3-phosphate dehydrogenase. Click here for file

Additional file 6: Figure S6 Showing effects of IHH-mediated or PTHrP-mediated signaling on the mRNA expression of *GREM1*, *FRZB* and *DKK1*. Chondrocytes were exposed to 2.5 μg/ml IHH, 10 μM cyclopamine, or 5×10^–7^ M PTHrP for up to 96 hours. At the indicated time points, mRNA expression was analyzed by quantitative PCR of *GREM1* (A), *FRZB* (B), *DKK1* (C), *GLI1* (D-E) and *CCND1* (F). Data expressed as fold-change relative to control and represent the mean of three donors ± standard deviation. *P <0.05 compared with untreated time point-matched controls. GADPH, glyceraldehyde 3-phosphate dehydrogenase. Click here for file
