# Supplementary figures and images for: GREM1, FRZB and DKK1 mRNA levels correlate with osteoarthritis and are regulated by osteoarthritis-associated factors
Source: Arthritis Res Ther. 2013 Sep 19;15(5):R126. doi: 10.1186/ar4306 (PMC3978825; doi:10.1186/ar4306)

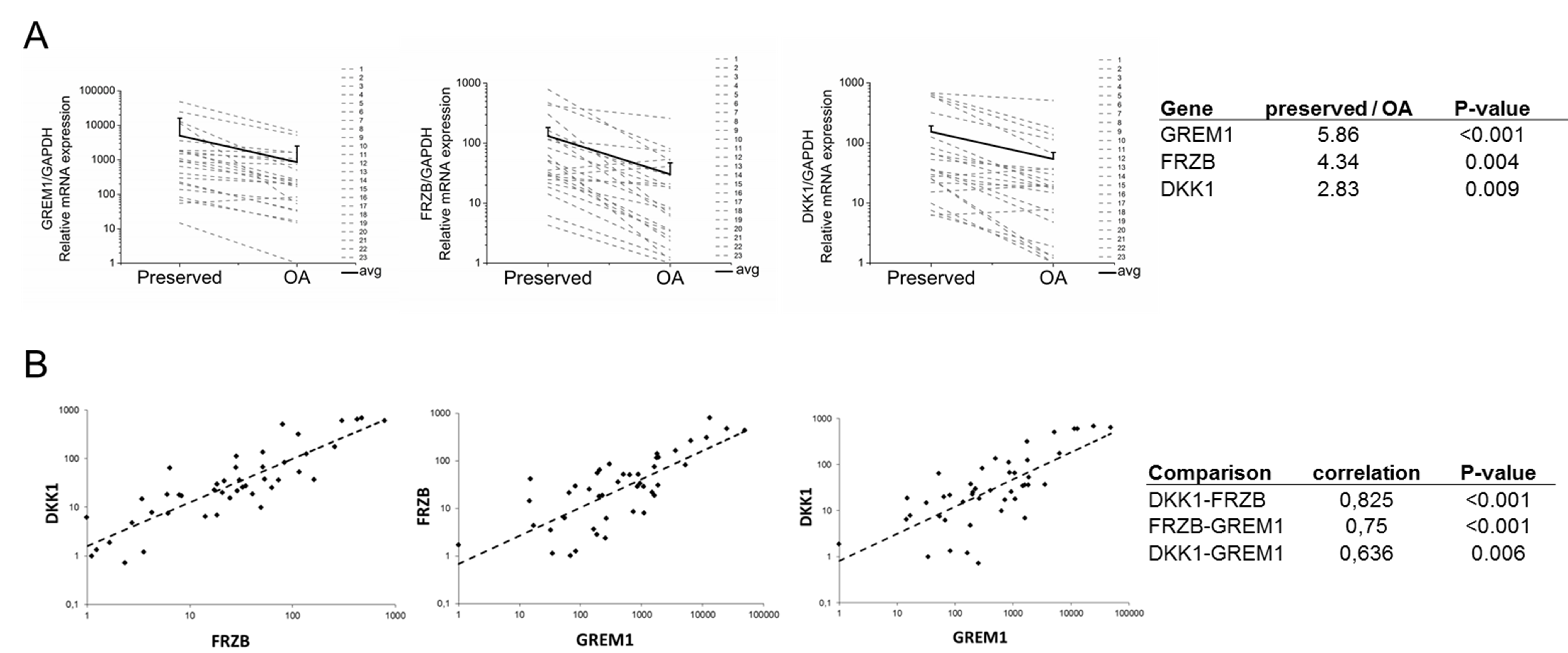

Supplement: Additional file 1: Figure S1 — Showing correlation of GREM1, FRZB and DKK1 mRNA expression in preserved and degrading cartilage from the same osteoarthritic joint (OA). (A) GREM1, FRZB and DKK1 mRNA levels were determined in preserved and degenerated cartilage from the same joint. All three antagonists are significantly less expressed in degenerated cartilage compared with preserved cartilage of the same joint (n = 23). Broken line indicates the cartilage specimen that belongs to the same donor. (B) GREM1, FRZB and DKK1 were investigated on correlation of mRNA expression. Correlation was determined using Pearson correlation. GADPH, glyceraldehyde 3-phosphate dehydrogenase. [file ar4306-S1.tiff]

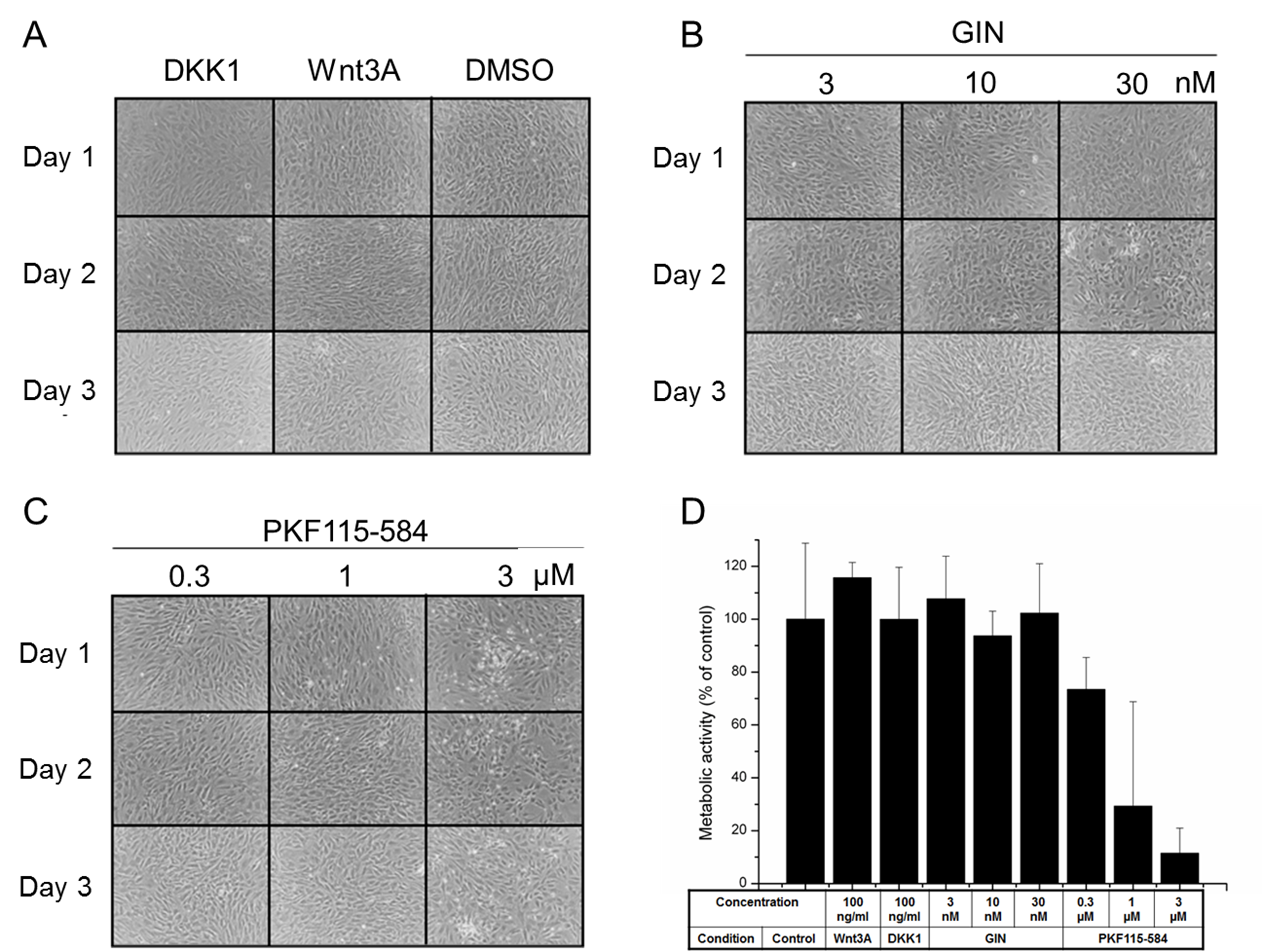

Supplement: Additional file 2: Figure S2 — Showing that cytotoxicity was phenotypically investigated by assessing chondrocyte morphology. Cells were exposed to DMSO, 100 ng/nl WNT3A, and 100 ng/ml DKK1 (A), a concentration range of GIN (B) and a concentration range of PKF115-584 (C). A representative picture of each condition over time is shown. To further investigate cytotoxicity, the metabolic activity of chondrocytes exposed to the different conditions was determined using Alamar Blue quantification (D). Data represent the mean of three donors ± standard deviation. DMSO, dimethylsulfoxide. [file ar4306-S2.tiff]

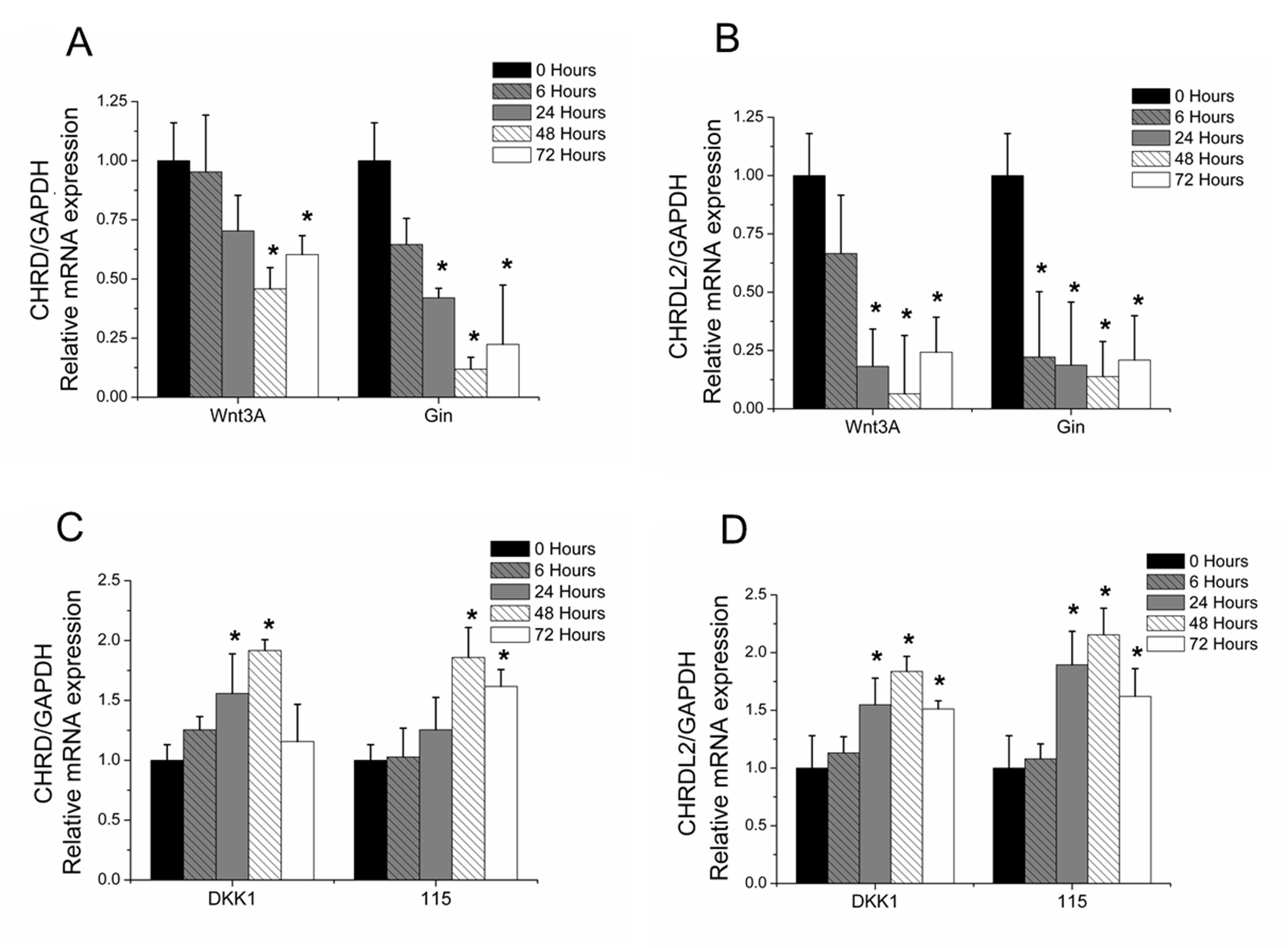

Supplement: Additional file 3: Figure S3 — Showing that WNT signaling decreases chordin and chordin-like 2 mRNA expression in primary human chondrocytes. Gene expression was measured by quantitative PCR. Chondrocytes were exposed to a single dose of 10 nM GIN, 100 ng/ml WNT3A (A), (B), 1 μM PKF115-584 or 100 ng/ml DKK1 (C), (D) for up to 72 hours and analyzed for the gene transcription of CHRD (A, C) and CHRDL2 (B, D). Data represent the mean of three donors ± standard deviation. *P <0.05 compared with untreated time point-matched controls. GADPH, glyceraldehyde 3-phosphate dehydrogenase. [file ar4306-S3.tiff]

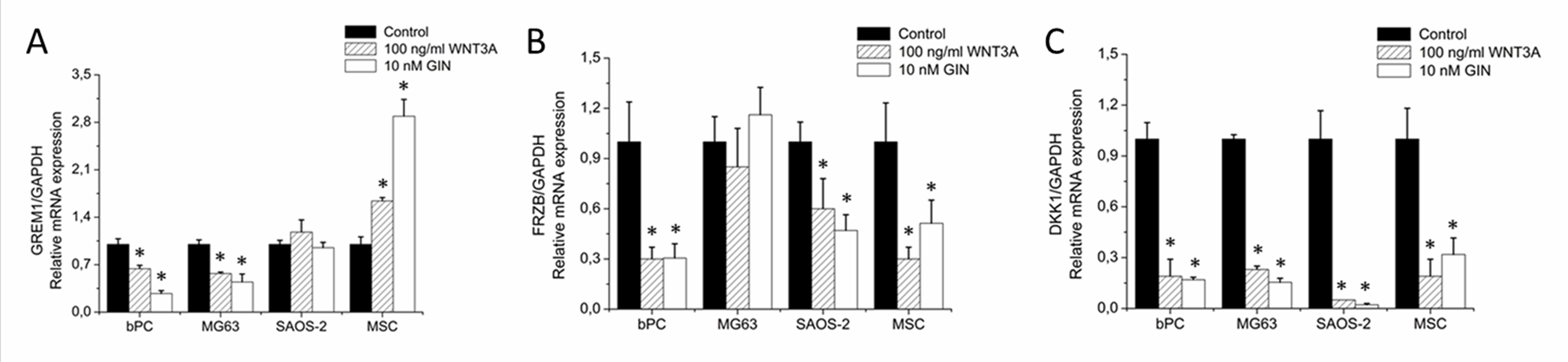

Supplement: Additional file 4: Figure S4 — Showing effects of activation of canonical WNT signaling on the mRNA expression of GREM1, FRZB and DKK1 in cells other than human chondrocytes. A single dose of 100 ng/ml WNT3A or 10 nM GIN was added to the culture media of bovine chondrocytes, the MG63 cell line, the SAOS-2 cell line and human bone marrow-derived MSCs. After 48 hours the gene transcription levels of GREM1 (A), FRZB (B) and DKK1 (C) were analyzed. Data represent the mean of three independent experiments (bovine chondrocytes, MG63 and SAOS-2) or two donors (MSCs) ± standard deviation. *P <0.05 compared with untreated time point-matched controls. GADPH, glyceraldehyde 3-phosphate dehydrogenase. [file ar4306-S4.tif]

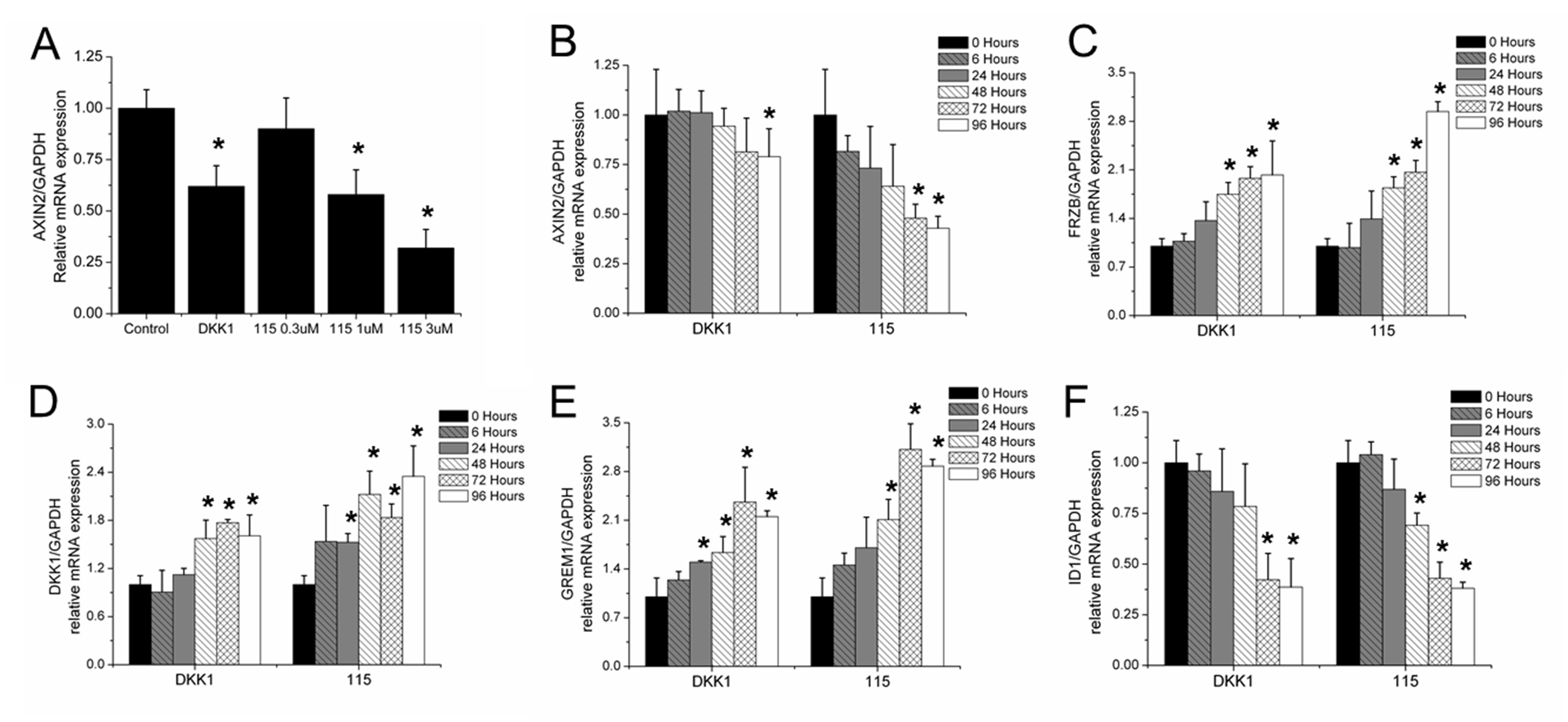

Supplement: Additional file 5: Figure S5 — Showing effect of canonical WNT signaling on the mRNA expression of GREM1, FRZB and DKK1. Primary human chondrocytes were exposed to 100 ng/ml WNT3A or three different concentrations of PKF115-584 (115). After 48 hours AXIN2 mRNA expression was analyzed by quantitative PCR. (A) Primary human chondrocytes were then exposed to 1 μM PKF115-584 or 100 ng/ml DKK1 for up to 96 hours. At indicated time points, gene expression was analyzed by quantitative PCR of AXIN2 (B), FRZB (C), DKK1 (D), GREM1, (E) and ID1 (F). Data expressed as fold-change relative to control and represents the mean of three donors ± standard deviation. *P <0.05 compared with untreated time point-matched controls (G) or 0 hours of stimulation (A to F). GADPH, glyceraldehyde 3-phosphate dehydrogenase. [file ar4306-S5.tiff]

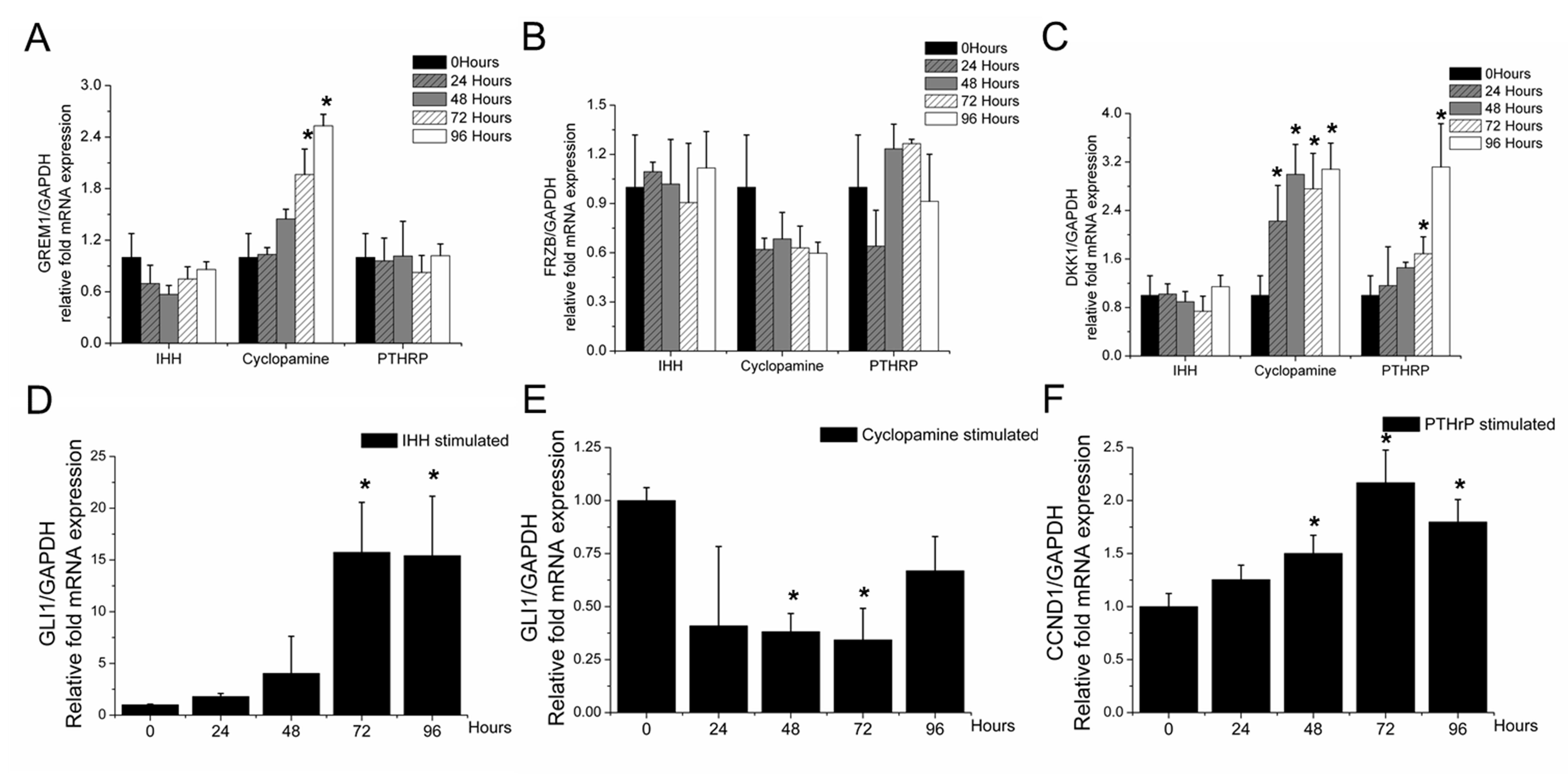

Supplement: Additional file 6: Figure S6 — Showing effects of IHH-mediated or PTHrP-mediated signaling on the mRNA expression of GREM1, FRZB and DKK1. Chondrocytes were exposed to 2.5 μg/ml IHH, 10 μM cyclopamine, or 5×10^–7^ M PTHrP for up to 96 hours. At the indicated time points, mRNA expression was analyzed by quantitative PCR of GREM1 (A), FRZB (B), DKK1 (C), GLI1 (D-E) and CCND1 (F). Data expressed as fold-change relative to control and represent the mean of three donors ± standard deviation. *P <0.05 compared with untreated time point-matched controls. GADPH, glyceraldehyde 3-phosphate dehydrogenase. [file ar4306-S6.tiff]
